# Tai chi for health benefits in patients with multiple sclerosis: A systematic review

**DOI:** 10.1371/journal.pone.0170212

**Published:** 2017-02-09

**Authors:** Liye Zou, Huiru Wang, ZhongJun Xiao, Qun Fang, Mark Zhang, Ting Li, Geng Du, Yang Liu

**Affiliations:** 1 Department of Exercise Science, Physical Education, and Wellness, Tennessee Tech University, TN, United States of America; 2 Department of Sport Science, Shanghai Jiaotong University, Shanghai, China; 3 Department of Foreign Language Teaching, Jishou University, Hunan, China; 4 Department of Physical Education and Health Education, Springfield College, MA, United States of America; 5 Department of Sport Management, Delaware State University, Dover, Delaware, United States of America; 6 Department of Neurology, Renji Hospital, Shanghai Jiaotong University, Shanghai, China; 7 Department of Exercise Science, Wuhan Sport University, Wuhan, China; 8 Department of Knesiology, Sensorimotor Neurophysiology Lab, Indiana University, Bloomington, Indiana, United States of America; National Natural Science Foundation of China, CHINA

## Abstract

The aim of this systematic review was to evaluate the existing evidence on the effectiveness and safety of Tai chi, which is critical to provide guidelines for clinicians to improve symptomatic management in patients with multiple sclerosis (MS). After performing electronic and manual searches of many sources, ten relevant peer-reviewed studies that met the inclusion criteria were retrieved. The existing evidence supports the effectiveness of Tai chi on improving quality of life (QOL) and functional balance in MS patients. A small number of these studies also reported the positive effect of Tai chi on flexibility, leg strength, gait, and pain. The effect of Tai chi on fatigue is inconsistent across studies. Although the findings demonstrate beneficial effects on improving outcome measures, especially for functional balance and QOL improvements, a conclusive claim should be made carefully for reasons such as methodological flaws, small sample size, lack of specific-disease instruments, unclear description of Tai chi protocol, unreported safety of Tai chi, and insufficient follow-up as documented by the existing literature. Future research should recruit a larger number of participants and utilize the experimental design with a long-term follow-up to ascertain the benefits of Tai chi for MS patients.

## Introduction

Multiple sclerosis (MS) is a progressive neurodegenerative disease, which may start as early as 20 years old and is caused by the immune system collapsing the protective myelin sheath in the central nervous system [1]. Demyelination interrupts communication between the central nervous system and effectors, resulting in a variety of symptoms (e.g. impaired balance and coordination, loss of sensation, pain, and fatigue) [2]. Multiple symptoms adversely from MS affect daily activities (e.g. work capability, socialization with others), which then lowers patients’ quality of life [3, 4].

The MS patient population has reached approximately 2.3 million worldwide and 300,000 in the U. S [5].The prevalence of MS patients requires costly investments and will eventually challenge the national healthcare system at some level. The cure for MS is still unknown. Additionally, MS has a fluctuating nature, causing the symptomatic management to be more challenging as the disease progresses [6]. Pharmacological management has been proven to alleviate the frequency and intensity of disease activity for patients with relapsing-remitting multiple sclerosis (RRMS) [7, 8], but pharmacological use has side effects.

Exercise as an alternative therapy has not only been recommended to healthy individuals [9, 10], but also individuals with chronic diseases [11, 12]. However, health professionals usually suggest MS patients to avoid exercises due to the heat and fatigue intolerance of the disease, but in 1999, Petajan and White indicate that certain types of mind-body exercises could be practiced by MS patients for health benefits, and Tai Chi (TC) is recommended [13].

Tai chi was originally created as a combating style in the Chinese martial art system, involving balance, strength, flexibility, speed, coordination, and agility. Over time, Tai chi has evolved into a multiple-element form of exercise containing gracefulness, mindfulness, softness, and gentleness that can be practiced by people across all ages. A great number of studies have shown the beneficial effects of Tai chi on physical as well as psychological well-being in both healthy older adults [14, 15] and patients with Parkinson’s diseases [16–18], but more recently attention has been paid to examining Tai chi for health benefits in patients with MS [19–21]. Therefore, carrying out this review to evaluate the existing evidence on the effectiveness and safety of Tai chi is critical in providing guidelines for clinicians to improve symptomatic management in MS patients.

## Methods

### Registration

This systematic study was registered with (PROSPERO) for two main reasons: (1) avoid occurrence of unplanned duplication of systematic reviews; (2) and demonstrate the transparent review process for minimizing bias of studies [22].

### Search strategy

Five electronic databases (Google scholar, PubMed, Physiotherapy Evidence Database [PEDro], and Cochrane Library) were utilized for literature search by entering the following terms in multiple combinations: “multiple sclerosis,” “demyelinating disease,” “Tai chi,” “Tai chi Chuan,” “Tai ji,” “Tai chi Chuan,” “Tai chi/Qigong,” “mind-body Qigong,” “Chinese mind-body exercise,” “traditional Chinese exercise,” “mindfulness-based exercise,” and “Tai ji Qigoing.” After conducting the electronic search, manual searches were subsequently performed through reference lists of the relevant publications.

### Eligibility criteria

Studies were included in this review if they met the following criteria: 1) peer-reviewed studies were published between 1985 and April, 2016; 2) participants aged 18 years or above, diagnosed with MS according to applicable diagnostic criteria [23, 24]; 3) Tai Chi was used as an exercise intervention for MS patients; 4) studies must include at least one of health outcome measures (e.g., balance, strength, mobility, flexibility, pain, fatigue, depression, or quality of life. To gain a comprehensive understanding about the effectiveness of Tai Chi in MS patients, there were no restrictions about types of studies. However, review articles, conference abstracts, magazine articles, monographs, and videos were excluded.

### Study selection

Two review authors (LYZ and HRW) independently performed screening process, involving two phases for obtaining eligible studies. Prior to the beginning of the screening process, the review authors first named three folders for all retrieved studies: “relevant”, “possibly relevant”, and “irrelevant”. In phase 1, the two review authors independently examined the titles and abstracts of all retrieved studies and placed them into the differently named folders. Based on percent agreement in two-rater model, the inter-rater reliability about the eligible studies within the relevant folder was calculated [25]. In phase 2 a third author emerged to discuss disagreement with the first two review authors so that they can reach an agreement about the eligible studies.

### Data extraction

For each eligible study, two review authors (LYZ and HRW) utilized pre-determined summary tables to independently extract the important information. Table 1 and Table 2 assess the methodological quality of experimental studies and observational studies, respectively. Table 3 includes author name and year of publication, study purpose, study design, place of study, sample (attribution%), age of patients, disease duration, and stage in disease progression. Table 4 includes author name and year of publication, intervention frequency and duration, outcome measures, results, conclusion, adverse events/follow-up, and effect size. A third party (ZJX) appeared to ensure that all detailed information was extracted.

**Table 1:** Study quality assessment for experimental studies.

Study	EC	RA	CA	SAB	SB	TB	AB	ITA	PMV	OSQ
Azimzadeh et al., (2015)	Yes	No	No	Yes	CD	No	CD	No	Yes	Low
Azimzadeh et al., (2013)	Yes	No	No	Yes	CD	No	CD	No	Yes	Low
Burschka et al., (2014)	Yes	No	No	Yes	CD	Yes	CD	No	Yes	Low
Kaur et al., (2014)	Yes	Yes	Yes	Yes	CD	Yes	CD	NA	Yes	Acceptable
Mills and Allen (2000)	Yes	Yes	Yes	Yes	Yes	Yes	Yes	NA	Yes	High
Mohali et al., (2013)	Yes	No	No	Yes	CD	No	CD	Yes	Yes	Low
Tavee et al., (2011)	Yes	No	No	Yes	CD	CD	Yes	NA	Yes	Low

Note: EC = eligibility criteria; RA = random allocation; CA = Concealed allocation; SAB = similar at baseline; SB = subject blinded; TB = Therapist blinded; AB = Assessor blinded; ITA = intention-to-treat analysis; PMV = Points measures and variability; OSQ = overall study quality = cannot determine; NA = not applicable.

**Table 2:** Study quality assessment for observational studies.

Study	RQ	ECSP	SPRC	EPE	SZ	ICD	OMCD	BOA	FUR	SA	MOM	GIIL	OSQ
Husted et al., 1999	Yes	Yes	Yes	Yes	No	Yes	Yes	CD	Yes (100%)	Yes	Yes	No	Acceptable
Mills et al., 2000	Yes	Yes	Yes	Yes	No	Yes	Yes	Yes	No (67%)	Yes	yes	Yes	High
Emmerik et al. 2014	Yes	Yes	Yes	Yes	No	Yes	Yes	CD	No (58.4%)	Yes	Yes	Yes	Acceptable

RQ = Research Question; ECSP = Eligibility criteria and study population; SPRC = study participants’ representative of clinical population of interest; EPE = all eligible participants enrolled; SZ = sample size; ICD = intervention clearly described; OMCD = outcome measures clearly described; BOA = blinding of outcome assessors; FUR = follow-up rate; SA = statistical analysis; MOM = multiple outcome measures; GIIL = group-level interventions and individual level outcome efforts; OSQ = overall study quality; NR = not reported; CD = cannot determine.

**Table 3:** Summary of TC for patients with multiple sclerosis (study purpose, study design, place of study, sample size (attribution%), age of patients, disease duration, and stage in disease progression.

Author, year	Study purpose	Study design	Place of study	Sample size (attrition%)	Age (year)	Disease duration	Stage in disease progression
Azimzadeh et al., 2015	To investigate the effect of TC on balance performance in female MS patients in Iran.	Quasi-experimental study	Tehran, Iran	• TC: 18/16 (11.1%) • CG: 18/18(0%)	Age ranging from 20 to 60 years old	Disease duration ranging from less than 6 years to more than 10 years.	EDDS score was smaller than 5
Azimzadeh et al., 2013	To assess the effect of TC on quality of life in women with MS	Quasi-experimental	Tehran, Iran	• TC: n = 16/16 (0%) • CG: n = 18/18(0%)	Female MS aged between 20 and 60 years old	Not reported	EDSS score is smaller than 5
Burschka et al., 2014	To explore the therapeutic value of TC for coordination, balance, fatigue and depression in mildly disabled MS patients	Quasi-experimental	Klinikum Bayreuth,Germany	• TC: n = 15/9 (40%) • CG: n = 17/17(0%)	• TC: 42.6(9.4) • CG: 43.6(8.0)	• TC: 6.0 (4.7) • CG:7.8 (6.8)	EDSS score was smaller than 5
Husted et al., 1999	To explore effectiveness of TC on psychological and physical benefits in MS patients	pretest/posttest	San Francisco, US	TC: 19/19 (0%)	Not reported	Not reported	Chronic progressive MS (n = 5), relapsing-remitting MS (n = 11), unknown type of MS (n = 4)
Kaur et al., 2014	To examine effectiveness of a combined exercise (TC and mindful practice) Vs TC on balance, gait, and mobility in patients with MS	RCT	Khajpura, India	• TCMP: n = 8/8 (0%) • TC: n = 8/8 (0%)	• TCMP: 36.75 (5.57) • TC: 36.75(8.31)	• TCMP: 7.25(3.10) • TC: 9.25(3.20)	• EDSS score for two groups: • TCMP: 3.06(1.76) • TC: 3.31(1.0)
Mills and Allen 2000	To investigate the effect of mindfulness-based TC on balance and symptoms in MS patients	RCT	South Wales, UK	• TC: 12/8 (33.3%) • CG: 12/8 (33/3%)	• TC: 48.6 (6.6) • CG:51 (7.0)	• TC: 21.6(4.3) • CG: 17.1(9.0)	• Secondary progressive, • ADL score • TC: 11(10.7) • CG: 17.2(11.5)
Mills et al., 2000	To explore the usefulness of TC as a pilot study on depression and balance	pretest/posttest	Wales, UK	TC: 12/8(33%)	Age ranging from 42 to 56	Year of diagnosis: ranging between 1972 and 1980 (study was conducted in 2000)	Secondary progressive; ADL score ranging from 1 to 28
Mohali et al., 2013	To examine the effectiveness of TC on balance in female MS patients	Quasi-experimental study	Mashhad, Iran	• TC: 15/15(0%) • CG: 15/15(0%)	Female MS patients aged between 30 and 40 years	Not reported	Not reported
Tavee et al., 2011	To determine the effect of TC on pain and quality of life in patients with MS.	Quasi-experimental design	Cleveland, US	• TC: n = 19/10 (52.6%) • CG: n = 11 /7 • (36.4%)	• TC:48.10(10.26) • CG:49.29(12.09)	• TC:10.4(6.47) • CG:19.14(14.35)	• EDSS for two groups: • TC: 3.25 (2.20) • CG: 2.79(2.80)
Emmerik et al. 2014	To examine effect of TC on balance and mobility in patients with MS	pretest/posttest	Amherst, Massachusetts, US	TC: 12/7(41.6%)	48.5(10.8)	Not reported	• EDSS score: 3.86(1.88), • PDDS: 2.42 (1.51)

RCT = randomized controlled trial; EDSS = Expanded Disability Status Scale; TC = Tai chi group; CG: control group; TCMP = Tai chi and mindful practice; ADL = The Activities of Daily Living Questionnaire.

**Table 4:** Summary of Tai Chi for patients with multiple sclerosis (intervention frequency and duration, outcome measures, results, conclusion, and adverse events/follow-up).

Author, year	Intervention frequency and duration	Outcome measures	Results	Conclusion	Adverse events	Effect size
Azimzadeh et al., 2015	• TC: two 45 to 60-minute group-based sessions weekly for 12 weeks. • CG: usual services including psychological lessons and physical therapy	BBS	MS patients in the TC group demonstrated a significant improvement on average balance scores between pre- and post-test, whereas no significant changes was observed in the control group.	TC could be taken into account as a safe complementary intervention to maintain and improve balance in MS patients	NR	0.15
Azimzadeh et al., 2013	• TC: Two sessions weekly for 12 weeks except keep regular healthcare • CG: usual treatment	MSQOL-54 scale	MS patients experiencing 12-week TC training has shown a significantly improvement on some subscales of quality of life (pain, emotional well-being, energy, social function, and health distress, overall quality of life, physical health composite score, mental health composite score), and total quality of life score (p < 0.05)	TC could be an alternative exercise intervention to improve quality of life in patients with MS	NR	NA
Burschka et al., 2014	• TC: Two 90-minute sessions weekly for 6 months • CG: usual treatment	Balance, coordination, fatigue (FSMC), Depression (CES-D), quality of life (QLS)	When compared to control group, MS patients in TC group demonstrated a significant improvement in balance (p = 0.031), coordination (p = 0.003), and depression (p = 0.007), quality of life (p = 0.012). In addition, MS patients in the control group had fatigue deterioration, whereas patients in TC group alleviated fatigue symptom (mean of pretest = 51.23 and mean of post-test = 47.6) even if not statistically significant finding (p = 0.182)	TC holds a promise as a therapeutic exercise for alleviating MS symptoms	NR	Balance: 0.79; Coordination: 0.83; QOL: 1.24
Husted et al., 1999	TC: two 1-hour sessions weekly for 8 weeks	Quality of life (SF-36), functional balance and mobility (walking distance = 25 ft) and flexibility	Subscales of the SF-36 were associated with significant improvements after 8-week TC training, including vitality, social functioning, mental health, and ability to perform physical and emotional roles. In addition, walking speed at the post-intervention test was 21% higher than the baseline walking speed; post-intervention flexibility was 28% greater than the baseline flexibility performance.	TC is useful to maximize independence and improve quality of life for patients with MS.	NR	NA
Kaur et al., 2014	• TCMP: twenty 60-minute sessions (20-minute mental practice, followed by 40-minute TC within 10–20 weeks. • TC: twenty 40-minute TC sessions within 10–20 weeks.	DGI, FRLF, TUG, and ASBC	Both intervention groups demonstrated significant improvement in balance, gait, and mobility in MS patients. No significant difference was observed between groups although the TCMP group performed better than the TC group.	TC is beneficial for improving balance and functional mobility in relapsing-remitting MS patients even if mindful-practice did not show statistical improvement in all tests.	NR	• DGI: 0.17 • FRF:0.3 • FRL: 1.20 • TUG:0.55 • ASBC: 1.02
Mills and Allen 2000	• TC: Each MS patient from the mindfulness-based group was given six individual one-to-one sessions, as well as provided written handouts, an audiotape, and a videotape for 3-month home-based practice as follow-up. • CG: keep usual care.	Symptom Rating Questionnaire, and single leg stand balance test	MS patients in the TC group were not associated with significant improvement on fatigue of the Symptom Rating Questionnaire. In addition, a significant improvement in balance performance was observed between pre- and post-test. Balance performance was observed to maintain after 3-month follow-up in five MS patients of the TC group, p < 0.05.	In addition to improving MS patient’s balance, mindful-based TC could be considered as a method to help patients with MS effectively perform self-symptom management because of improved physical and psychological domains. In contrast, MS patients tended to deteriorate in symptoms.	NR, but 3-follow-up	Balance: 1.48
Mills et al., 2000	After six individual TC sessions, MS patients were encouraged to perform at least 30-minute home-based TC practice per day for 2 months, guided by a videotape with audio-taped instructions	POMS, Check-list of physical symptoms, and Balance (single le-standing test)	Significant improvements were observed in depression dejection between pre (6.25) and post measure (3.00) (p < 0.04) and in fatigue-inertia between pre (13.88) and post measure (11.25) (p < 0.03) in terms of the POMS; a significant improvement was observed on balance between pre- (5.63) and posttest (11.88) (p < 0.05). In addition, other symptoms in MS patients were reported to gain improvement, including spasms, numbness, bladder control and walking.	Authors concluded that TC not only helps MS patients to alleviate depression and other symptoms (spasms numbness, bladder control and walking), but also strengthens physical balance.	NR	NA
Mohali et al., 2013	• TC: three sessions weekly for 8 weeks • CG: usual care	BBS	MS patients in the TC group were associated with a significant improvement in mean points of balance (p < 0.001), whereas those in the control group did not demonstrate a significant change from baseline to post-intervention test.	Authors concluded that TC could be viewed as an alternative exercise to improve balance in MS patients and lower frequency of falling in their daily lives.	NR	NA
Tavee et al., 2011	• TC: three 30-minute sessions weekly for 2 months • CG: usual treatment	SF-36, VAS, and MFIS-5	MS patients in the intervention group arm demonstrated a significant improvement in scores for pain (p = 0.031), and fatigue (p = 0.035). In addition, after the 2-month intervention, the combined TC intervention group (MS and patients with peripheral neuropathy) demonstrated an improvement in summed physical health scores on the SF-36 (p = 0.011 MS, p = 0.014 PN), summed mental health scores (p = 0.02), vitality (p = .005), and physical role (p = .003).	Mindfulness-based TC may be helpful in reducing pain and improving quality of life in patients with MS.	NR	NA
Emmerik et al. 2014	TC: three 1-hour TC session for 3 weeks	postural stability, leg strength (a chair rise test), and neural drive, psychosocial wellbeing (Multiple Sclerosis Impact Scale-29), Fatigue Severity Scale.	Significant improvement in leg strength (p = 0.024), neural drive (rapid foot tapping) (p = 0.025), dynamic balance (p = 0.02), total psychosocial well-being (p = 0.032) were observed. Static balance measured using tandem stance showed no significant improvement (p = 0.66) but increased static balance control. No change in general fatigue or leg specific fatigue severity score.	TC intervention can potentially improve multiple functional systems (somatosensation, neural drive, strength and balance) and reduce fear of falling.	NR	NA

MSQOL-54 scale = Multiple Sclerosis Quality of Life Questionnaire; SF-36 = 36-item Short Form Health Status Survey; VAS = a visual analogue scale for pain; PDDS = Patient-determined Disease Steps Questionnaire; MFIS-5 = 5-item Modified Fatigue Impact Scale; CES-D = Center for Epidemiological Studies Depression Scale; FSMC = Fatigue Scale of Motor and Cognitive Function; QLS = Questionnaire of Life Satisfaction; DGI = Dynamic Gait Index measuring the mobility function and the dynamic balance; FRLF = Functional Reach test including lateral (FRL) and forward (FRF) directions; TUG = Time Up and Go; ASBC = Activities-specific Balance Confidence; BBS = Berg Balance Scale; POMS = Profile of Mood States; NR = not reported.

### Assessment of risk bias

The methodological quality of all eligible studies was systematically evaluated using Delphi quality criteria for assessment of experimental trials [26] and observational studies [27, 28]. With regard to the quality criteria for assessment of experimental studies, it included nine questions assessing risk bias of the eligible studies: random assignment, concealment of allocation, similar baseline, eligibility criteria, blinding of outcome assessor, blinding of subject, blinding of therapist, points measures and variability, and intention-t-treat analysis. With regard to the quality criteria for assessment of observational studies, quality assessment tools were determined based on types of observational studies. For example, quality assessment tool for uncontrolled, pretest/posttest design, included 12 questions to examine four types of risk bias for each eligible study: research question, eligibility criteria and study population, study participant representative of clinical populations of interest, all eligible participants enrolled, sample size, intervention clearly described, outcome measures clearly described, blinding of outcome assessors, follow-up rate, statistical analysis, multiple outcome measures, and group-level interventions and individual-level outcome efforts. For the experimental studies, if a study met seven to nine criteria, overall quality of the study was considered to be high, indicating little or no risk of bias; if a study met four to six criteria, overall quality of the study was considered to be acceptable, indicating some risk of bias; if a study only met one to three criteria, the overall quality of the study was considered to be low. For the observational studies, if a study met nine to twelve criteria, overall quality of the study was considered to be high with little or no risk of bias; if a study met five to eight criteria, overall quality of the study was considered to be acceptable with some risk of bias; if a study only met one to four criteria, overall quality of the study was considered to be poor. Two review authors (LYZ and MZ) independently performed the methodological quality assessment using the same quality assessment tools. A third review author (ZJX) calculated the interrater reliability about the results of the study quality assessment.

### Data synthesis

Initially, review authors (LYZ and QF) intended to use Revman 5.3 software within the Cochrane Collaboration for data synthesis [29]. Due to the small number of randomized controlled studies, and the heterogeneity of outcome measures in the eligible studies, a meta-analysis was not performed. Therefore, the review authors (LYZ, ZJX, GD, and QF) carried out a qualitative synthesis based on the eligible studies. For those experimental studies, if mean and standard deviation were reported, effect sizes (Cohen’s d) were calculated based on the standardized formulas. A value of the effect size represents the magnitude of TC intervention that is interpreted as follows: 0.2 = small, 0.5 = medium, and 0.8 = large [30].

## Results

### Study selection

**Fig 1:**
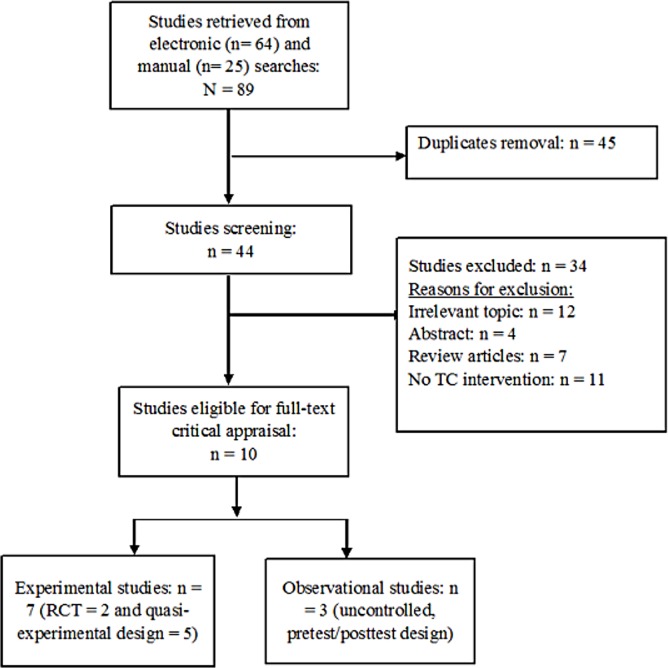
Flow chart showing the study selection.

A total of 89 studies were retrieved from both electronic (n = 64) and manual (n = 25) searches. Only 44 studies remained after removing 45 duplicates based on titles and abstracts. This was followed by using eligible criteria to exclude 34 articles due to the following reasons: irrelevant topic (n = 12), abstract (n = 4), review articles (n = 7), and no TC intervention (n = 11). Ten studies were finally considered eligible for full-text critical appraisal, including seven experimental studies (two randomized controlled design [31, 32] and five quasi-experimental design [19–21, 33, 34]) and three observational studies (-posttest design [35–37]). The inter-rater reliability for the selection of the eligible studies was 83.3%. The flowchart showing the study selection for this systematic review is presented as below (Fig 1).

### Methodological quality assessment for eligible studies

The inter-rater reliability for methodological quality assessment within the ten eligible studies was 90%. A third party emerged to resolve a disagreement between the first two review authors (LYZ and HRW) about one eligible study. The methodological quality assessment for experimental studies and observational studies are presented in Table 1 and Table 2, respectively. With regard to the study quality assessment for seven experimental studies, of the two studies, one with acceptable [31] quality and another one with high quality [32] were presented, whereas the remaining five eligible studies demonstrated low overall study quality [19–21, 33, 34]. More specifically, the low overall study quality within the experimental studies is mainly attributed to the following reasons such as selection bias (non-randomized, unconcealed assignment), performance bias (absence of blinding of therapist), measurement bias (absence of blinding of participants and assessor), and attrition bias (lacking intention-to-treat analysis). With regard to the study quality assessment for three observational studies with pretest-posttest design, although one study with high quality [36] and two other studies with acceptable quality [35, 37] were presented, all three studies lacked large sample size [35–37]; two studies lacked blinding of outcome assessor [35, 37]; one study did not meet the requirement of “group-level interventions and individual-level outcome efforts” [35].

### Study characteristics

The characteristics of the ten eligible studies are presented in Table 3 and Table 4. These eligible studies were conducted in five different countries (Iran, Germany, USA, India, and UK) and then published between 1999 and 2015, using three main designs (randomized controlled, quasi-experimental, and pretest-posttest) to examine the effect of TC on various health outcomes (e.g., balance, coordination, mobility, flexibility, strength, fatigue, depression, pain, and QOL) in MS patients. Study sample size ranged from 12 to 36 and attrition percentage ranged from zero to 52.6. Study participants ranged in age between 20 and 60 with an average disease duration ranging from less than six to 21.6 years. With regard to the stage of disease progression, study participants presented either Expanded Disability Status Scale (EDSS) score of less than five or Activities of Daily Living Questionnaire (ADL) score ranging from one to twenty-eight. With regard to the intervention frequency and duration, TC intervention sessions ranged from 30 to 90 minutes for two or three sessions weekly in most of the studies when the TC intervention program ranged from two to six months. For the health outcome measures, a variety of reliable and valid assessment tools were utilized, including Multiple Sclerosis Quality of Life Questionnaire, 36-item Short Form Health Status Survey, a visual analogue scale for pain, Patient-determined Disease Steps Questionnaire, 5-item Modified Fatigue Impact Scale, Center for Epidemiological Studies Depression Scale, Fatigue Scale of Motor and Cognitive Function, Questionnaire of Life Satisfaction, Dynamic Gait Index, Functional Lateral-Forward Reach test, Time Up and Go, Activities-specific Balance Confidence, Berg Balance Scale, and Profile of Mood States.

## Summary of evidence

Table 4 also presents a summary of the study results, conclusion, adverse event, and effect size associated with health outcomes. For the 10 eligible studies, we classified outcome measures into quality of life, physical function (balance, gait, mobility, flexibility, and leg strength, and coordination), fatigue, and other MS symptoms (depression and pain).

### Tai chi for quality of life

Recently more attention has been paid to QOL assessment in MS patients because it covers multiple dimensions of life (physical wellbeing, material wellbeing, social wellbeing, emotional wellbeing, development, and activity). QOL assessment can better evaluate disease progression and symptomatic management when compared to assessment of fatigue, depression, or physical disability, which only reflects a small portion of life experience in MS patients [38–40]. Five studies examining the effect of TC on QOL in MS patients were included in this review [20, 21, 33, 35, 37].

Azimzadeh, Hosseini, and Tabrizi [33] examined the effect of TC on QOL in 34 female MS patients, recruited from the Multiple Sclerosis Society. With a non-equivalent, randomized, and controlled trial, the 34 MS patients either received TC training (n = 16) or usual treatment (n = 18). The researchers used the Multiple Sclerosis Quality of Life Questionnaire (MSQOL-54 scale), which is a reliable and valid disease-specific QOL instrument [38]. After a 12-week intervention period, MS patients in the TC group demonstrated significant improvements on subscales of quality of life such as pain, emotional well being, energy, social function, health distress, overall quality of life, physical health composite score, and mental health composite, as well as total quality of life (*p* < 0.05). However, the researchers did not report disease duration and stage in disease progression.

Tavee, Rensel, Planchon, Butler, and Stone [21] explored if an 8-week meditation-based TC was more effective for QOL in MS patients by using a prospective, nonrandomized, and controlled trial. To obtain relatively large statistical power, researchers combined two different types of patients, MS and peripheral neuropathy (PN). 39 participants (19 with MS and 17 with PN) volunteered to participate in the meditation-based TC and 25 patients (11 with MS, 14 with PN) were assigned to the control group. The final analysis included 22 (10 MS, 12 PN) in the Tai chi group and 18 (7MS, 12 PN) in the control group after 21 withdrew from the study because of unavailable transportation, loss of interest, hospitalization, and deteriorating inflammation. For the MS and PN patients combined, statistical analyses were used. Separate statistical analyses for only MS were also conducted. After 8 weeks, participants in the TCgroup showed significant improvements in SF-36 scores for overall summed physical health (*p* = .011 MS), mental health (*p* = .02 combined group), vitality (*p* = 0.005 combined group), and physical role (*p* = 0.003 combined group), whereas no improvement in the control was observed.

Burschka, Keune, Oy, Oschmann, and Kuhn [20] designed a randomized controlled study to explore if TC could be used as a therapeutic exercise to improve QOL (Questionnaire of Life Satisfaction) in mildly disabled MS patients (relapsing-remitting multiple sclerosis = 27, secondary progressive = 4, and clinically isolated syndrome = 1). After 6 withdrew from the study (schedule conflicts = 5 and health problem = 1), 32 MS patients (Expanded Disability Status Scale, EDSS < 5) were used for final analysis, including 15 in the TC group (two 90-minute sessions weekly for 6 months while keeping the usual healthcare) and 17 in the control groups (keeping usual healthcare). Although the researchers failed to report use of intention-to-treat analysis, a significant improvement on QOL was reported in the TC group (*p* = 0.012), with an increase of 16.8 QOL from mean score of baseline (215.77) to posttest (232.57), when compared to MS patients in the control group demonstrating a decrease of 10.65 QOL from mean score of baseline (204.46) to posttest (193.81) (*p* = 0.29).

Long-term healthcare for MS patients is needed in order to improve the quality of their lives. However, expensive medicine restricts the majority of MS patients to meet the need for symptomatic management. Searching for affordable therapeutic exercises is essential for MS patients to ease their economic burden. Therefore, Husted, Pham, Hekking, and Niederman [35] examined the therapeutic effect of TC on improving QOL in 19 MS patients, with a pretest-posttest design. The MS patients experienced two 1-hour sessions weekly for 8 weeks. The QOL was measured at baseline and after the 8-week intervention, using a generic Medical Outcomes Study 36-item Short-form Health Survey. Improvements were observed on the subscales (vitality, social function, mental health, and ability to perform physical and emotion roles) of the SF-36.

Emmerik, Jones, Busa, Remeius, and Averill [37] carried out a pretest-posttest trial examining the effect of TC on Multiple Sclerosis Impact Scale-29 (MSIS-29) measuring QOL, with a small sample size of 12 MS patients (age 48.5 ±10.8 years, height 1.66 ±0.08 m, mass 68.6 ±19.8 kg). The MS patients experienced nine 1-hour TC sessions within three weeks. Only seven MS patients were included for final analysis after five withdrew from the study. The total psychosocial wellbeing of the MSIS-29 was associated with a significant improvement (*p* = 0.032).

### Tai chi for physical function

Impaired balance and mobility are two main symptoms of MS patients. More than 90% of MS patients were reported to have balance dysfunctions [41, 42]. The detrimental effect of balance dsyfunction was associated with significantly higher frequency of falling while performing daily activities, which worsens the social and economic burdens in patients [43, 44]. Therefore, researchers should focus more on the usefulness of TC for improving balance, mobility (dynamic balance), coordination, flexibility, and leg strength, because these abilities are important for people to perform voluntary locomotion movements.

Burschka et al. [20] examined a six-month TC training program versus usual treatment on balance and coordination in MS patients. Balance tests (static and dynamic) consisted of 14 tasks with an order of increasing difficulty. For the static balance assessment, MS patients were asked to perform single leg stances in different conditions (descriptions of the different conditions were not reported); the dynamic balance was measured by walking forward, making a turn, and walking back toward the start line on a wooden floor. Similarly, a coordination test consisted of 10 tasks, with an order of increasing difficulty. For the balance and coordination tests, MS patients were awarded one point if he or she completed each task. A maximum of 14 points for the balance test and 10 points for the coordination test can be given to each MS patient. MS patients were asked to complete the two tests at baseline and after a six-month intervention. Significant improvements on both balance (p = 0.031; effect size = 0.79) and coordination performance (p = 0.003; effect size = 0.83) were observed in the TC group, whereas slightly decreased mean scores emerged in the group receiving usual treatment, from pretest (6.88) to posttest (6.53) in the balance test, and from pretest (4.94) to posttest (4.82) in the coordination test.

Researchers examined the effectiveness of a combined exercise (TC and mindful practice) versus TC on balance, gait, and mobility in 16 patients with RRMS, with a randomized, controlled, paralleled study [31]. Eight RRMS patients in Group 1 experienced twenty 60-minute sessions (20-minute mental practice, followed by 40-minute TC) over 10 to 20 weeks. Eight in Group 2 completed twenty 40-minute TC sessions over the same duration. The study included Dynamic Gait Index (DGI) (measuring mobility and dynamic balance), Functional Balance Reach (FR) test at lateral and forward directions, Timed Up and Go (TUG) (measuring gait speed, dynamic balance and mobility), and Activities-specific Balance Confidence (ASBC). The method for data analysis was not reported. Although both intervention arms demonstrated significant improvements in DGI (effect size = 0.17), FRF (effect size = 0.3), FRL (effect size = 1.2), TUG (effect size = 0.55), and ASBC (effect size = 1.02), no significant differences existed between the two groups. Researchers concluded that TC is beneficial for improving balance and functional mobility in RRMS patients.

Non-equivalent control, pretest-posttest design was used in two different studies, examining the effectiveness of TC on balance, as measured by the Berg Balance Scale in female MS patients [19, 34]. Azimzadeh, Hosseini, Nourozi, and Davidson [19] equally assigned 36 MS patients into two groups: an intervention group experiencing two 45 to 60-minute group-based TC sessions weekly for 12 weeks and the control group receiving counseling sessions and physical therapy as usual services. Mohali, Ebrahimi, Hassan, Khoshraftar, and Shoeibi [34] recruited 30 MS patients from the MS Society and assigned them into two groups: an intervention group receiving an 8-week TC training with three sessions weekly and the control group only receiving usual treatment. Although both studies did not report the statistical significance, improvements (effect size = 0.15) on average balance scores between baseline and post-test were observed in both (p = 0.003) [19] and (p < 0.001) [34], whereas no significant changes were observed in the control groups.

Two research groups conducted similar studies about the usefulness of TC on balance performance in secondary progressive MS patients with two different study designs: pretest-posttest [36] and randomized-controlled-paralleled design [32]. Mills, Allen, and Carey-Morgan [36] initially assigned 12 MS patients into intervention training receiving six individual TC sessions, followed by 30 minutes of home-based TC exercises guided by a videotape per day for the rest of the 2-month intervention period. Four withdrew because of bereavement, loss of interest, or incomplete symptom diaries. The results of the final analysis including the eight MS patients showed a significant improvement on balance performance between baseline (5.63) and posttest (11.88) (p < 0.05). Mills and Allen [32] utilized mindfulness-based TC to help with symptom management in eight MS patients. Balance assessment was performed at baseline, after six TC training sessions, and a 3-month follow-up. Although the description of statistical analysis was not reported, the TC group was associated with a significant improvement on balance performance (effect size = 1.48), and five MS patients still maintained this balance performance at the 3-month follow-up assessment.

Apretest-posttest design was used in two different studies, examining the effectiveness of short-term TC training on lower limb-related functions, including balance (static balance and mobility), neural drive, flexibility, and leg strength [35, 37]. Husted et al. [35] used 25 feet walking distance (measuring functional balance and mobility) and hamstring flexibility tests as outcome measures in the study. After an 8-week TC training, walking speed of MS patients was 21% faster than the baseline walking speed and post-intervention flexibility was 28% greater when compared to the baseline flexibility performance. In the study of a 3-week TC training for benefits in balance, gait, leg strength, and the neural drive was conducted by [37]. MS patients were asked to perform multiple tests, including sensorimotor (plantar sensation and toe taps measuring neural drive), Chair Rise test (leg strength), and average center of pressure velocity (COP) and total excursion and time to contact (TTC) for postural stability. Multiple significant improvements were observed in plantar sensation (*p* = .02), neural drive (*p* = 0.024), leg strength (*p* = 0.025), and dynamic balance (*p* = 0.022). Postural sway velocity of Tandem Stance test did not demonstrate significant improvement, but significant improvements in the Tandem Stance test on TTC at Antero-Posterior (*p* = .005) and Medio-lateral (*p* = .05) directions were observed.

### Tai chi for fatigue

Fatigue is defined as a subjective perception of physical or mental energy deficiency while performing voluntary movements in daily life [45]. Fatigue is one of the most commonly reported symptoms in patients with MS, affecting roughly four-fifths of MS patients [46]. Patients with multiple sclerosis do not only experience fatigue, but multiple motor functions may also be deteriorated by MS-related fatigue, including balance and mobility [47, 48]. TC has been proven to provide fatigue relief in patients with breast cancer [49] and rheumatoid arthritis [50], therefore it may be an alternative method for fatigue management in MS patients.

Five studies [20, 21, 32, 36, 37]were found that examined the effect of TC on fatigue in patients with MS. Fatigue was measured using three different assessment tools, including the 5-item Modified Fatigue Impact Scale (MFIS-5) [21], the Fatigue Scale of Motor and Cognitive Function (FSMC) [20], the Fatigue Severity Scale (FSS) [37], the Profile of Mood States (POMS) [36], and the Symptom Rating Questionnaire (SRQ) [32]. The results of the five studies were not consistent. Of those, TC training was proved to have a significant beneficial effect on fatigue relief in two studies [21, 36], but this benefit was not observed in three studies [20, 32, 37].

### Tai chi for depression and pain

Two studies assessed the effectiveness of TC on depression [20, 36]. Burschka et al. [20] used the Center for Epidemiological Studies Depression (CES-D) scale, reporting a significant reduction in depression (*p* = 0.007) in MS patients in the TC group, in comparison to the control group. A significant reduction was also observed in depression as measured by the POMS between pre-test (6.25) and post measure (3.00; *p* < 0.04) in [36]. Pain is a relatively common symptom, following MS patients about half of the time over the course of their disease [50]. Pain management is highly reliant on conventional medicine, but side effects are often unavoidable. Tavee et al. [21] conducted a study examining if TC is beneficial for pain relief in patients with MS, and reported a significant reduction in pain (*p* = 0.031), as measured by the Visual Analogue Scale for Pain (VAS).

## Discussion

The main aim of this review was to evaluate the evidence of TC for MS. The existing evidence supports the effectiveness of TC on improving the quality of life and functional balance in MS patients. A small number of studies reported the therapeutic effect of TC for MS patients on coordination, flexibility, leg strength, gait, and pain. The findings of studies examining the effect of TC on fatigue were inconsistent.

There are a variety of systematic reviews evaluating the effectiveness of TC for older adults [51], postmenopausal women [52], patients with rheumatoid arthritis [53], patients with cardiovascular diseases [54], and patients with Parkinson’s disease [55]. To date, limited studies have been done to examine the efficacy of TC for MS patients. TC is beneficial, particularly for functional balance and QOL among MS patients, which is consistent with a large number of studies investigating TC for patients with other neurological disorders such as Parkinson’s disease [16, 56, 57]. The positive findings could be explained by the principle of TC focusing not only external physical function improvements (e.g. weight shifting movements for improving postural control, leg strength, flexibility, and coordination), but also strengthening internal energy (e.g. vitality, bodily pain, and fatigue) in order to obtain higher quality of life [58].

Although the existing evidence tends to support the beneficial effect of TC for the above-mentioned outcome measures, making a definitive claim is still limited because of the number of studies with methodological flaws (e.g. lack of randomized controlled and paralleled trials, unavailable blinding of assessors, unclear statistical analysis), and small sample size. In addition, this paper includes more generic QOL assessment tools, instead of disease-specific QOL instruments, which could negatively affect the interpretation of the positive findings. Using the most appropriate QOL measure is critical for clinicians to better evaluate disease progression and symptomatic managements. Freeman, Hobart, and Thompson [59] emphasized that when compared to the general QOL instruments that focus on QOL measure in a different population, disease-specific QOL instruments are more appropriate because they are purposefully designed to examine exact health problems and are more sensitive for detection and quantification of change scores from baseline to post-intervention test.

MS-related fatigue is a subjective disabling symptom with unknown pathogenesis, affecting roughly 80% of MS patients in their daily activities such as work and social capabilities [60–62]. Whether is beneficial for fatigue relief still remains uncertain because of the inconsistent findings of this systematic review,which is consistentwith conclusions of a systematic review examining efficacy of multiple interventions for MS-related fatigue, including pharmacological (amantadine, modafinil, and pemoline) and non-pharmacological (behavioral advice, cooling vests and electromagnetic fields, acupuncture, yoga, cannabis, and bee venom) interventions [63]. The different findings in this systematic review may be attributed to variations in TC dosage per session across studies; 30 to 90-minute TC session is actually equivalent to a light to high-intensity exercise, requiring practitioners to release a different amount of internal energy (heat) in order to efficiently perform TC movements. For instance, MS patients participating in a 60- or 90-minute TC session are more likely to produce a substantial amount of heat, which could aggravate fatigue symptom [20, 37], as opposed to a 30-minute Tai chi session showing significant improvement in terms of fatigue [21, 36]. Other confounding factors may also affect fatigue assessment, including MS type, disease duration, stage in disease progression, frequency of MS relapses, and duration and frequency of healthcare use, but they were not controlled for in the studies of this systematic review.

The unclear description of training protocol including specific TC movements is a limitation. Taking into account the complex symptomatic features of MS, MS patients may have more difficulty performing TC movements in comparison to the normal population. TC movements that are appropriate for the healthy population may not be the best fit for MS patients. Therefore, the most appropriate individualized TC protocol should be created based on the baseline assessment of the individual, rather than designing an exercise protocol prior to the beginning of the research project. Peer-reviewed articles examining the effectiveness of TC for MS patients or even other diseases should include a detailed description of TC protocol or videotape. The existing evidence only stated: “TC used as an intervention.” This general information would not be helpful enough for clinicians to retrieve exact data. Because Wang, Yu, Chen, Lu, and Yu [64] found that even the same type of TC with two different versions (simplified versus traditional TC) had different impacts on slowing bone loss in postmenopausal women. Choosing more appropriate TC movements for MS patients should be taken into account in future studies.

None of the studies reported adverse events or new symptoms, which may emerge during the TC intervention period. Achiron, Barak, Stern, and Noy [65] found an MS patient experiencing electrical sensation during Tai chi exercise and suggested that physicians pay more attention to symptoms or events that occur while attending exercise sessions. Although TC has been proven to be a safe alternative exercise for patients with other neurological disorders [16, 57], the safety of TC for MS patients still remains unknown. MS is fluctuating and often progressive which may potentially affect assessing the continued effect of TC on improving functional balance and QOL. If post-intervention follow-up is added in future studies, the long-term effect of TC for MS patients would be identified. However, only one study in this review used a 3-month post-intervention follow-up [32].

## Conclusions

The existing evidence demonstrates a significant beneficial effect of TC on health outcomes in MS patients, especially for functional balance and QOL improvements. However, a conclusive claim should be made carefully because of methodological flaws, small sample size, lack of specific-disease instruments, unclear description of Tai chi protocol, an unreported safety of TC, and insufficient follow-up. Future studies with a larger number of study participants, high-quality experimental design (e.g. randomized controlled trial, rigorous statistical analysis, specific-disease instruments, clear description of TC protocol, and available report of adverse events), and a follow-up should be applied to test the long-term effectiveness of TC for MS patients.

## Supporting information

S1 Appendix PRISMA 2009 Checklist. (PDF)
